# Efficacy of immunotherapy in *KRAS*-mutant advanced NSCLC: A real-world study in a Chinese population

**DOI:** 10.3389/fonc.2022.1070761

**Published:** 2023-01-19

**Authors:** Lixiu Peng, Jun Guo, Li Kong, Yong Huang, Ning Tang, Juguang Zhang, Minglei Wang, Xiaohan He, Zhenzhen Li, Yonggang Peng, Zhehai Wang, Xiao Han

**Affiliations:** ^1^ Department of Medical Oncology, Shandong Cancer Hospital and Institute, Shandong First Medical University and Shandong Academy of Medical Sciences, Jinan, China; ^2^ Department of Radiation Oncology, Shandong Cancer Hospital and Institute, Shandong First Medical University and Shandong Academy of Medical Sciences, Jinan, China; ^3^ Department of Imageology, Shandong Cancer Hospital and Institute, Shandong First Medical University and Shandong Academy of Medical Sciences, Jinan, China; ^4^ Department of Medical Science, Berry Oncology Corporation, Beijing, China; ^5^ Department of Bioinformatics, Berry Oncology Corporation, Beijing, China

**Keywords:** NSCLC, *KRAS*, immunotherapy, co-mutation, *KRAS*-mutant subtypes

## Abstract

**Background:**

Immunotherapy has improved the clinical outcomes of patients with advanced non-small cell lung cancer (NSCLC). However, in patients with Kirsten rat sarcoma viral oncogene homolog (*KRAS*) mutations, the superior efficacy of immunotherapy has not been elucidated and especially in real-world practice. Our study aimed to use real-world data to assess the efficacy of immunotherapy in *KRAS*-mutant NSCLC in a Chinese cohort.

**Methods:**

In this retrospective cohort study, we extracted the clinical, molecular, and pathologic data from the electronic health records of patients with advanced *KRAS*-mutant NSCLC at Shandong Cancer Hospital between January 2018 and May 2022. Furthermore, we evaluated the progression-free survival (PFS) and overall survival (OS) of the included patients.

**Results:**

Between January 2018 and November 2020, 793 patients were identified with stage IIIB-IV NSCLC and a total of 122 patients with *KRAS* mutations were included in the analysis. The majority of patients were diagnosed with stage IV (82.0%) adenocarcinoma (93.4%), along with a history of smoking (57.4%). Of these, 42% of patients received anti-PD-(L)1 with or without chemotherapy (Immunotherapy-based regimens), while 58.2% of patients received chemotherapy (Chemotherapy-based regimens). The median overall survival (mOS) in this cohort was 22.9 months (95% CI: 14.1–31.7), while the median-progression-free survival (mPFS) was 9.4 months (95% CI: 6.6–12.1). Patients receiving immunotherapy-based regimens displayed better mOS than those receiving chemotherapy-based regimens (45.2 vs. 11.3 months; *P*=1.81E-05), with no statistical difference observed in the mPFS (10.5 vs. 8.2 months; *P*=0.706). Patients receiving immunotherapy-based regimens either in the first line (*P*=0.00038, *P*=0.010, respectively) or second-line setting (*P*=0.010, *P*=0.026, respectively) showed benefits in both PFS and OS. Subgroup analysis indicated that in patients having *KRAS* G12C or non-*KRAS* G12C mutant types, immunotherapy showed benefits of better OS (*P*=0.0037, *P*=0.020, respectively) than chemotherapy. Moreover, in advanced NSCLCs patients with or without *KRAS/TP53* co-mutation the immunotherapy-based regimen achieved longer OS and PFS than chemotherapy-based regimens.

**Conclusions:**

In the Chinese population of patients with *KRAS*-mutant advanced NSCLC, immunotherapy-based regimens achieved longer OS than chemotherapy-based regimens, which was independent of first or second-line setting, as well as *KRAS* mutational subtypes.

## 1 Introduction

Non-small cell lung cancer (NSCLC) remains one of the major causes of cancer-related deaths in China and worldwide (1). The most common oncogenic driver in NSCLC is the mutation of Kirsten rat sarcoma viral oncogene homolog (*KRAS*), exhibiting approximately 20–30% prevalence among Western countries and 10–15% among Asian countries (2). *KRAS* mutant NSCLC is considered a heterogeneous disease regarding *KRAS* mutant subtypes, co-mutations (3), and immunogenic profiles (4). Biological heterogeneity is suggested to play a role in the vulnerability to therapy, tumor microenvironment, and immune modulatory effects. For instance, patients with *KRAS/TP53* co-mutations were reported to be sensitive to immunotherapy (Objective Response Rate[ORR]: 35.7%), while patients with *KRAS/STK11* displayed poorer outcomes upon treatment with immunotherapy (ORR: 7.4%) (5). However, a retrospective study showed that *KRAS*-mutant NSCLC might benefit from chemo-immunotherapy (6). *KRAS* has long been considered ‘undruggable’ (7), and the management of *KRAS*-addicted lung cancer is considered the same as that of non-oncogene-addicted cancer (8). Furthermore, limited treatment options and high heterogeneity may increase the difficulties of managing advanced *KRAS*-mutant patients.

Research on optimal management of *KRAS*-mutant NSCLC is still in progress. However, a breakthrough was achieved in the treatment landscape when the US Food and Drug Administration (FDA) approved direct *KRAS* G12C inhibitor Sotorasib for advanced or metastatic NSCLC adult patients having *KRAS* G12C local mutation, with patients receiving one prior systemic therapy. Immunotherapy is considered promising cancer therapy. Although most oncogene-addicted tumors, including *EGFR*-or *ALK*-driven lung cancer, do not respond to immunotherapy (9), even at >50% of PD-L1 expression. However, this is not the case in *KRAS* mutant NSCLC. The response rate to immunotherapy in such patients is shown to be at least the same or even better than that of *KRAS*-wild type patients (10–13). Few studies have also confirmed the superior efficacy of immunotherapy over chemotherapy in the *KRAS*-mutant NSCLC population. For instance, in one meta-analysis including three clinical trials, Kim et al. showed the superior efficacy of immunotherapy over chemotherapy in *KRAS*-mutant patients in the second-line setting (14). Similarly, a recent meta−analysis including six randomized controlled trials with 386 *KRAS*-mutant NSCLC patients suggested that anti-PD-(L)1 with or without chemotherapy displayed a significant association with prolonged OS (HR=0.59, 95%CI: 0.49–0.72; P<0.00001) and PFS (HR=0.58, 95%CI:0.43–0.78; *P*=0.0003) compared to chemotherapy alone (15).

However, since these findings were from the subgroup analysis of clinical studies, validating them in a real-world setting was necessary. Therefore, we conducted a real-world study in a Chinese population to verify the efficacy of immunotherapy with or without chemotherapy in *KRAS*-mutated advanced NSCLC patients.

## 2 Methods

### 2.1 Study design and data source

The data for this retrospective observational cohort analysis was extracted from the electronic health records of patients at Shandong First Medical University Cancer Hospital and Shandong Cancer Hospital. The study was approved by the Ethics Committee of Shandong First Medical University Cancer Hospital and Shandong Cancer Hospital. Between January 2018 and November 2020, the patient records with stage IIIB-IV NSCLC were included in the study. The cohort used in this study was based on 793 patients. The above-mentioned clinical information mainly included baseline characteristics (sex, age, smoking status, histological subtype, ECOG PS, and tumor stage), *KRAS* mutation status, and treatment history of the patients. Furthermore, the patients were followed up from the date of diagnosis till the date of death due to all causes or up to the latest available follow-up.

### 2.2 Cohort selection

Initially, patients included in the cohort met the following inclusion criteria: Age 18 years or older; diagnosed with stage IIIB to stage IV NSCLC with evidence of mutation in *KRAS*; receiving treatments from diagnosis to the end of follow-up. The exclusion criteria included records with no adequate information of pathological diagnosis, evidence of mutation in *EGFR* or *ALK* gene arrangement and *ROS1* translocation, and records of EGFR TKIs treatment. The chemotherapy-based regimen was defined as the non-addition of anti-PD(L) 1 in the management of patients during the period of treatment.

### 2.3 Therapeutic regimens

Of the 51 immunotherapy-based patients, 6 received ICI monotherapy and 45 received ICI combination therapy with the following regimens: monotherapy: sintilimab, pembrolizumab, tislelizumab, and camrelizumab; combination therapy: sintilimab plus pemetrexed/platinum-based, sintilimab plus nab-paclitaxel/platinum-based, sintilimab plus docetaxel, pembrolizumab plus pemetrexed/platinum-based, tislelizumab plus pemetrexed/platinum-based, atelelizumab plus nab-paclitaxel/platinum-based, atelelizumab combined with bevacizumab and paclitaxel and platinum-based, toripalizumab combined with pemetrexed/platinum-based. Of the 71 patients treated with chemotherapy received the following conventional chemotherapy regimens: pemetrexed plus carboplatin or cisplatin, paclitaxel plus carboplatin or cisplatin, docetaxel plus carboplatin or cisplatin, gemcitabine plus carboplatin or cisplatin, bevacizumab combined with pemetrexed/platinum-based or paclitaxel/platinum-based.

Among the 122 patients, 24 patients were treated with first-line immunotherapy, 98 patients were treated with first-line chemotherapy, 21 patients were treated with second-line immunotherapy, and 26 patients were treated with second-line chemotherapy.

### 2.4 Study endpoints

The primary endpoint was OS, which was defined as the period starting from the diagnosis till death or the date of the last follow-up. The secondary endpoint was real-world progression-free survival (rwPFS), defined as the time from diagnosis until objective tumor progression or death, whichever occurs first. Our study used a clinician-anchored approach supported by radiology data. Based on the radiology scan and pathologic confirmation *via* tissue biopsy or through clinical assessment, the clinician-recorded assessment was used to determine disease progression. Patients with missing information regarding the date of the last clinical note and progression were excluded from the rwPFS analysis.

### 2.5 Molecular profiling

Amplification refractory mutation system-polymerase chain reaction (ARMS-PCR) was used to identify *KRAS* mutation status. Genomic alterations were detected in patient samples using targeted sequencing panels (BerryOncology, Beijing), including a 456-gene (BerryOncology, Beijing) and a 36-gene test panel (BerryOncology, Beijing).

### 2.6 Statistical analysis

Standard descriptive statistics were used to compare the cohort characteristics between the chemotherapy- and immunotherapy-based regimen groups. The Fisher’s exact test and Mann-Whitney Wilcoxon test were used to compare the differences among variables of both groups, which included age, gender, smoking history, clinical stage, *KRAS* mutation subtype, *KRAS* gene co-mutation, distant metastasis, and the presence or absence of radiotherapy. Kaplan–Meier analysis was performed to estimate the survival rate, while the log-rank test was performed to test the differences in survival distribution among the subgroups. Moreover, the Cox proportional hazard regression model was used for univariate analyses. All statistical analyses were performed using the SPSS version 23.0, IBM software. The difference was considered statistically significant if the *P*-value was less than 0.05.

## 3 Results

### 3.1 Clinical characteristics

**Figure 1:**
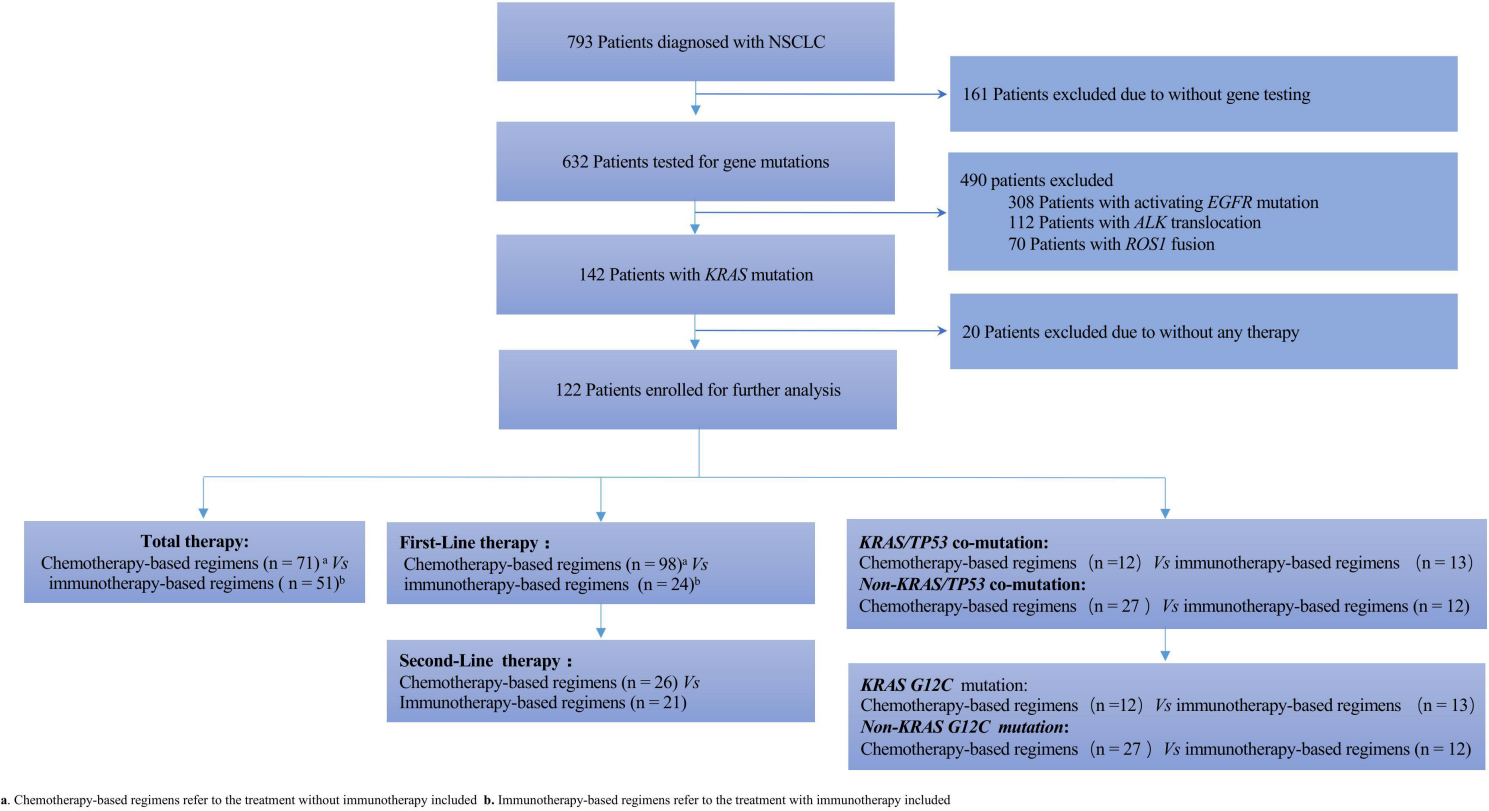
Flow chart depicting patient selection. NSCLC, non-small cell lung cancer.

Of 632 patients with available gene test results, *KRAS* mutation was identified in a total of 142 advanced NSCLC patients. Among them, 20 patients did not receive any treatment at our hospital. Hence, we finally included 122 patients in our retrospective analysis, as shown in **Figure 1**, whose detailed clinical characteristics are summarized in **Table 1**. The cohort comprised 100 (82.0%) males and 22 (18.0%) females having an average age of 62 years. The major histological subtype included adenocarcinoma (n=114, 93.4%). Of these, 100 (82.0%) patients had recurrent or stage IV disease at the time of diagnosis. Additionally, 57.4% of the patients had a history of smoking. Finally, all patients were treated based on their clinical staging status. Our results showed no significant differences in clinical characteristics, except for anti-angiogenesis therapy (*P*=0.03)

**Table 1:** The characteristics of KRAS-mutant NSCLC patient.

Characteristics	All N = 122(%)	Immunotherapy-based regimens N =51(%)	Chemotherapy-based regimens N = 71(%)	*P-value*
Gender				0.35
Male	100 (82.0)	44 (86.3)	56 (78.9)	
Female	22 (18.0)	7 (13.7)	15 (21.1)	
Age				0.55
<60	38 (31.1)	14 (27.5)	24 (33.8)	
≥60	84 (68.9)	37 (72.5)	47 (66.2)	
Smoking history	–	–	–	0.50
Smoker	70 (57.4)	27 (52.9)	43 (60.6)	
Never smoked	52 (42.6)	24 (47.1)	28 (39.4)	
Histological subtype				0.72
Adenocarcinoma	114 (93.4)	48 (94.1)	66 (93.0)	
Squamous	3 (2.5)	2 (3.9)	1 (1.4)	
Adenosquamous	3 (2.5)	1 (2.0)	2 (2.8)	
Other	2 (1.6)	0 (0)	2 (2.8)	
ECOG PS				0.21
0∼1	103 (84.4)	46 (90.2)	57 (80.3)	
2	19 (15.6)	5 (9.8)	14 (19.7)	
Staging				0.64
IIIB/IIIC	22 (18.0)	8 (15.7)	14 (19.7)	
IV	100 (82.0)	43 (84.3)	57 (80.3)	
*KRAS* mutant				0.20
G12C	25 (20.5)	13(25.5)	12 (16.9)	–
Non-G12C	39 (32.0)	12 (23.5)	27 (38.0)	
Unknown	58 (47.5)	26 (51.0)	32 (45.1)	
Co-mutations				0.24
KRAS/TP53	25 (20.5)	12 (23.5)	13 (18.3)	
NonKRAS/TP53	39 (32.0)	12 (23.5)	27 (38.0)	
Unknown	58 (47.5)	27 (53.0)	31 (43.7)	
Metastatic sites				0.15
Brain	30 (24.6)	17 (33.3)	13 (18.3)	
Liver	5 (4.1)	0 (0)	5 (7.1)	
Bone	27 (22.1)	10 (19.6)	17 (23.9)	
Other sites	35 (28.7)	13 (25.5)	22 (31.0)	
None	25 (20.5)	11(21.6)	14(19.7)	
Radiotherapy				0.14
Yes	66(54.1)	32 (62.7)	34 (47.9)	
No	56 (45.9)	19 (37.3)	37 (52.1)	
Anti-angiogenesis therapy				
Yes	57 (46.7)	30 (58.8)	27 (38.0)	0.03
No	65 (53.3)	21 (41.2)	44 (62.0)	

### 3.2 Immunotherapy-based regimens improved the survival outcomes of *KRAS*-mutant advanced NSCLC patients in both first-line and second-line settings

**Figure 2:**
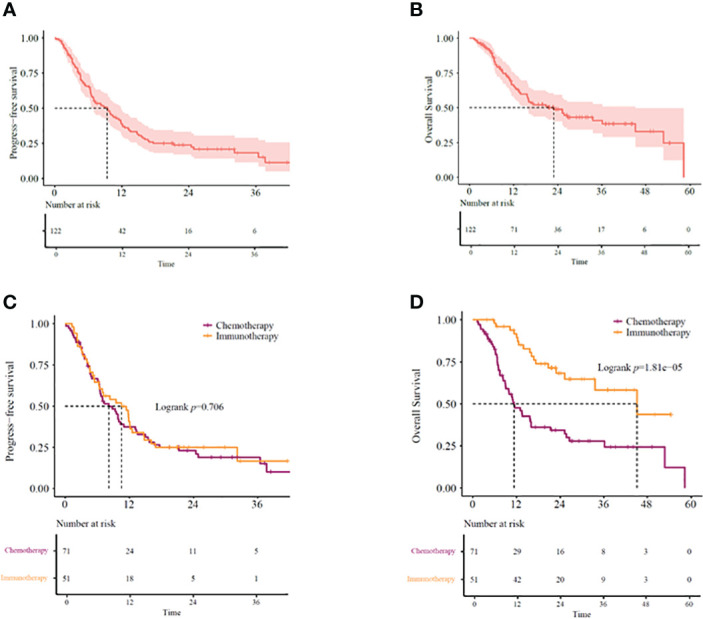
**(A)** PFS and **(B)** OS in *KRAS*-mutant advanced NSCLC patients. **(C)** PFS and **(D)** OS in *KRAS*-mutant advanced NSCLC patients receiving immunotherapy- or chemotherapy-based regimens. OS, overall survival; PFS, progression-free survival.

**Figure 3:**
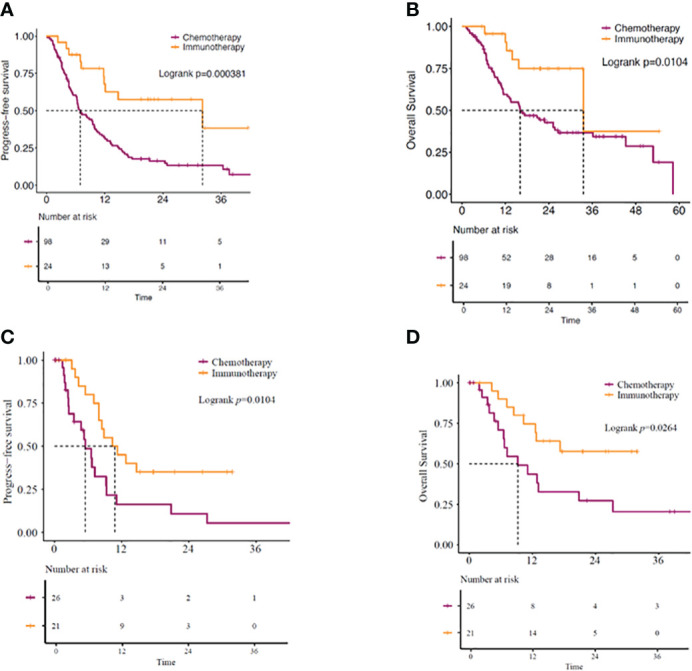
**(A)** PFS and **(B)** OS in *KRAS*-mutant advanced NSCLC patients. Patients receiving immunotherapy- or chemotherapy-based regimens as first-line of treatment. **(C)** PFS and **(D)** OS in *KRAS*-mutant advanced NSCLC patients receiving immunotherapy- or chemotherapy-based regimens as second-line of treatment. OS, overall survival; PFS, progression-free survival.

Our study showed the median overall survival (mOS) of *KRAS*-mutant advanced NSCLC patients as 22.9 months (95% CI: 14.07–31.67) and the median progression-free survival (mPFS) as 9.4 months (95% CI: 6.60–12.14) (**Figures 2A** **, B**). While 51 (41.8%) patients received immunotherapy-based regimens, 71 (58.2%) received chemotherapy-based regimens (**Table 1**). Patients receiving immunotherapy-based regimens displayed significantly longer mOS compared to patients receiving chemotherapy-based regimens (45.2 vs. 11.3 months; *P*=1.81E-5), with no significant difference observed in the mPFS (10.5 vs. 8.2 months; *P*=0.706) (**Figures 2C** **, D**). Additionally, immunotherapy from both first-and second-line treatments showed survival benefits. Patients receiving immunotherapy-based regimens as the first line of treatment displayed better mOS and mPFS than those receiving chemotherapy-based regimens (mOS: 33.5 vs. 16.1 months; *P=*0.010, mPFS: 32.2 vs. 6.9 months; *P=*0.00038) (**Figures 3A** **, B**). Similarly, the patients receiving immunotherapy as the second line of treatment also displayed significant improvement in the mOS and mPFS compared to those receiving chemotherapy (mOS: NR vs. 9.23 months; *P*=0.026, mPFS: 10.8 vs. 5.5 months; *P=*0.010) (**Figures 3C** **, D**).

### 3.3 Efficacy of immunotherapy in *KRAS* G12C and *KRAS* non-G12C subgroups

**Figure 4:**
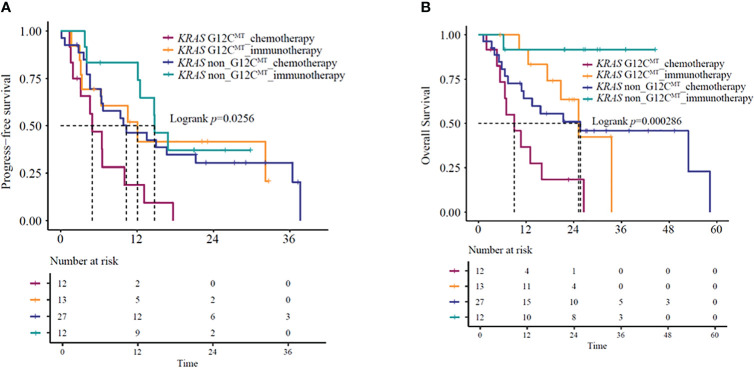
**(A)** PFS and **(B)** OS in *KRAS* G12C-mutant and *KRAS* non-G12C mutant NSCLC patients receiving immunotherapy- or chemotherapy-based regimens. OS, overall survival; PFS, progression-free survival.

Since specific *KRAS* mutational subtypes may exert different effects on treatment response and survival, we aimed to characterize the effects of *KRAS* mutation subtypes on the OS and treatment response of these patients. With the available information on mutation revealed by molecular characterization, we stratified the patients into *KRAS* G12C and *KRAS* non-G12C subgroups. Genomic profiles of 64 *KRAS* mutant patients were analyzed using next-generation sequencing (Berryoncology, Beijing), which detected two major mutation subtypes, including G12C (20.5%) and non-G12C (32.0%). The G12C status was unknown for 47.5% of the patients. Among the four different categories of *KRAS*-mutant NSCLCs, significant differences were observed in both mOS and mPFS (mOS: log-rank test, *P*=0.00020; mPFS: log-rank test, *P*=0.026) (**Figures 4A** **, B**). Further analysis revealed that *KRAS* G12C and non-G12C subtype patients treated with immunotherapy-based regimens showed significantly better mOS compared to the same patients receiving chemotherapy-based regimens (G12C group HR=0.23,95%CI:0.08-0.67, *P*=0.0074; mOS: 25.2 vs. 9.1 months, *P*=0.0037; non-G12C group HR=0.13,95%CI:0.02-0.99, *P*=0.049; mOS: NR vs. 25.7 months, *P*=0.020). However, significant difference for PFS was observed in G12C group but not in non-G12C group (G12C group HR=0.38,95%CI:0.14-0.99, *P*=0.047; mPFS: 12.1 vs. 5.0 months, *P*=0.039; non-G12C group HR=0.73,95%CI:0.3-1.75, *P*=0.48; mPFS: 14.8 vs. 10.3 months, *P*=0.48).

### 3.4 The impact of concurrent pathogenic mutations *KRAS/TP53* on the efficacy of immunotherapy

**Figure 5:**
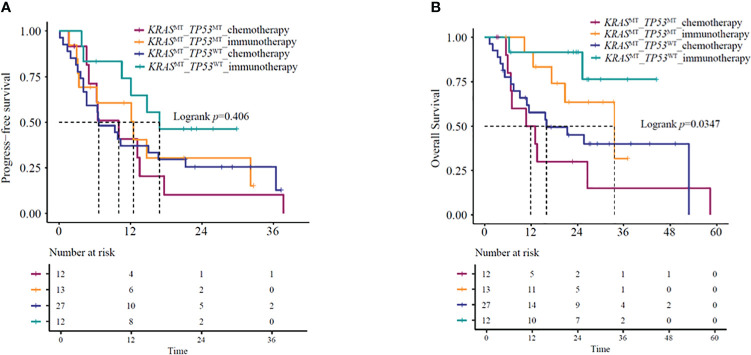
**(A)** PFS and **(B)** OS in *KRAS/TP53* co-mutation or *KRAS* mutant/*TP53* wild-type patients receiving immunotherapy- or chemotherapy-based regimens. OS, overall survival; PFS, progression-free survival.

Several studies (16–18) have indicated that under immunotherapy, the co-mutation status of advanced *KRAS*-mutant type exerts an impact on the patient’s clinical outcomes. Based on the co-mutation status, we used the NGS results of 64 patients for survival analysis. The identified co-mutations included *TP53* (20.3%), *PIK3CA* (1.6%), and *STK11* (0.8%). Kaplan-Meier curves based on *TP53* mutation status and treatment group showed a significant difference in mOS (*P*=0.035) (**Figure 5A**) but not in mPFS (*P* = 0.41) (**Figure 5B**). Further analysis suggested that *KRAS/TP53* co-mutation group and non- *KRAS/TP53* mutation group patients treated with immunotherapy-based regimens showed significantly better mOS compared to the same patients receiving chemotherapy-based regimens (*KRAS/TP53* co-mutation group HR=0.32, 95%CI:0.1-0.98, *P*=0.047; mOS:33.5 vs. 11.8 months, *P*=0.036; non-*KRAS/TP53* co-mutation group HR=0.23,95%CI:0.05-0.99, *P*=0.049; mOS: NA vs. 16 months, *P*=0.031). However, no significant difference was observed in the mPFS (*KRAS/TP53* co-mutation group HR=0.78,95%CI:0.31-1.96, *P*=0.59; mPFS:12.5 vs.10.0 months, *P*=0.59; non- *KRAS/TP53* co-mutation group HR=0.49,95%CI:0.2-1.23, *P*=0.13; mPFS: 16.9 vs. 6.7 months, *P*=0.12).

## 4 Discussion

*KRAS*-mutant NSCLC is a genetically heterogeneous disease with distinct biology and therapeutic vulnerabilities. An effective choice of treatment for this disease is immunotherapy. However, further investigation, especially in real-world settings, may be required to verify the efficacy of immunotherapy in *KRAS*-mutant NSCLC patients. Therefore, we retrospectively studied 122 advanced NSCLC patients with *KRAS* mutations for their prognosis and obtained the mOS at 22.9 months (**Figure 2B**). This result was similar to a previous study, where mOS was 28 months (19). Furthermore, the mOS was 25.8 months in the study of El Osta., et al, which was similar to our study (20). In our study, patients receiving immunotherapy-based regimes displayed a significantly longer OS than those receiving chemotherapy-based regimens (45.2 vs. 11.3 months, *P*=0.001) (**Figure 2D**). Moreover, the survival benefits were independent of whether it was the first-line setting or second-line setting, which was also consistent with the subgroup analysis results of previous clinical trials (15). In addition, outcomes of the KEYNOTE189 shows that patients receiving immunotherapy plus chemotherapy have longer mPFS than those receiving chemotherapy (9 vs. 5 months, HR=0.47,95%CI [0.29-0.77]) in *KRAS* -mutated lung cancer (21). In the 2022 ASCO meeting, data scientists from the FDA conducted a large retrospective analysis, including 555 metastatic NSCLC patients with *KRAS* mutations. Their analysis concluded that the chemo-immune checkpoint inhibitor combination produced the greatest survival benefit compared to the treatment with immune checkpoint inhibitors (ICIs) or chemotherapy alone and hence, should be given to such patients upfront (22). Specifically, chemo-ICIs as the first line of treatment were linked to a response rate of 46%, while ICI alone generated a response rate of 37%, indicating that chemo-immunotherapy may be the optimal management option for the advanced *KRAS*-mutant NSCLC patients both in white and Asian populations.

The enhanced survival benefits in this study can be explained using several biological rationales. *KRAS* mutations in NSCLC were associated with tobacco smoking, a high tumor mutational burden (TMB), and an inflammatory tumor microenvironment, along with high T-cell infiltration (23). Importantly, compared to the wild-type counterparts, *KRAS*-mutant tumors showed higher expression of PD-L1, with the median PD-L1 tumor proportion scores ranging between 30–60% and 5–35% in patients with and without *KRAS* mutations, respectively (21, 24). One study suggested that the activation of the *KRAS*-signaling pathway resulted in the inhibition of tristetraprolin activity, which is important for the stabilization of PD-L1-mRNA and, thus, its synthesis (25). Another study showed that *KRAS* mutations were correlated to an inflammatory tumor microenvironment and tumor immunogenicity, which benefitted the response to ICIs (23). Since *KRAS*-mutated NSCLC is typically smoking-related lung cancer, with more than 90% of patients having a history of smoking, it is more likely that such patients will respond to ICI treatment.

Notably, the patients treated with a combination of anti-PD(L)1 and chemotherapy (immunotherapy-based regimens) showed an mOS of 45 months, which was longer than most previous studies (21, 26). This may be because the Eastern Cooperative Oncology Group performance score (ECOG PS) of the patients was between 1 and 2. The value of ECOG PS was 0~1 in 84.4% of patients and 2 in 15.6% of patients. Multiple retrospective cohort studies across different tumor types have suggested that patients with ECOG PS ≥2 showed worse response rates, faster progression, and shorter OS (27–29). Additionally, a recent study showed that mOS of advanced NSCLC patients with good performance status was 30 months (95% CI 16.6–42.3), but in patients with poor performance status, it was only 4 months (95% CI 3.2–8.1) (30), which was similar to our results.

No significant difference was observed in PFS between immunotherapy and chemotherapy. Studies suggested no correlation between the mOS and mPFS (31, 32) in immunotherapy-related clinical trials. Moreover, in randomized clinical trials of PD-1 inhibitors, the effect of treatment was higher on OS than on PFS (31), which was consistent with our results. This suggested that PFS may not be able to capture the benefits of immune checkpoint inhibitors. PD-1 inhibitors have residual efficacy for a longer duration, and even after the discontinuation of treatment, these drugs could affect OS more than PFS. Therefore, the RECIST criteria may not be completely suitable to measure the immunotherapy response.

Previous studies demonstrated that *KRAS* G12C mutations and *TP53* co-mutations were correlated to benefits obtained from anti-PD-1/PD-L1 immunotherapy (18). Similar results were also found in this study, where patients with *KRAS-*G12C mutation receiving immunotherapy with or without chemotherapy achieved more survival benefits than chemotherapy alone. This indicated the significant role of immunotherapy in the clinical management of these patients. The combination strategy may abolish the adverse OS impact of the *KRAS* G12C mutant. A preclinical study suggested that *KRAS* G12C inhibition can swiftly change the tumor’s immune-suppressive microenvironment to the one that allows effective anti-tumor immunity (33). In addition, a phase 2 trial results of sotorasib for lung cancers with *KRAS* G12C mutation showed that the mPFS was 6.8 months (95% CI, 5.1 to 8.2) and the mOS was 12.5 months (95% CI, 10.0 to could not be evaluated) (34).Due to a higher level of PD-L1 expression, T cell infiltration, and tumor immunogenicity, the *KRAS/TP53* co-mutation in NSCLC exhibited sensitivity to anti-PD-1/PD-L1 immunotherapy (17). In our study, the advanced NSCLCs patients with or without *KRAS*/*TP53* co-mutation benefitted more from the immunotherapy-based regimens than chemotherapy-based regimens in mOS (*KRAS* ^MT^ *TP53* ^WT^ mOS: *P=*0.36; *KRAS* ^MT^ *TP53* ^WT^ mOS: *P=*0.049). Furthermore, no significant differences were observed in mPFS between immunotherapy receiving *KRAS* ^MT^ *TP53* ^MT^ and *KRAS* ^MT^ *TP53* ^WT^ patients (*KRAS* ^MT^ *TP53* ^MT^ mPFS: *P=*0.59; *KRAS* ^MT^ *TP53* ^WT^ mPFS: *P=*0.12), which may be due to the small size of our study sample. Hence, this aspect may require further investigation.

### 4.1 Limitations

The first limitation of our study was the insufficient characterization of the genomic profiles of the patients, with ARMS-PCR being applied to only nearly half of the patients. Also, performing survival analyses in subgroups based on *KRAS*-mutation and co-mutation status was challenging. Second, since heterogeneous patients with various levels of PD-L1 expression and TMB status, *KRAS* mutation status may have affected the survival outcomes of ICIs differently as per the expression level of PD-L1. For example, patients with high PD-L1 levels receiving immunotherapy as the first line of treatment may have fared as well as those who received chemo-immunotherapy (35). Also, these levels were only available in a small proportion of patients. Hence, whether the superior efficacy of ICIs observed in this study was independent of TMB status and/or PD-L1 expression remains unknown. Thirdly, our study was a single-center study and not fully representative of the broader population of cancer patients in China, which in some cases, may limit the generalizability of the obtained data. Therefore, to make informed clinical decisions, further studies may be needed to provide sufficient evidence.

## Data availability statement

The original contributions presented in the study are included in the article/supplementary material. Further inquiries can be directed to the corresponding authors.

## Ethics statement

The studies involving human participants were reviewed and approved by Ethics Committee of Shandong First Medical University Cancer Hospital and Shandong Cancer Hospital. The patients/participants provided their written informed consent to participate in this study.

## Author contributions

ZHW and XH designed the research and revised the article. LXP and JG analyzed the data and wrote the manuscript. ZZL analyzed the data. LK, YH, NT, JGZ and MLW collected the data. XHH and YGP revised the article. All authors contributed to the article and approved the submitted version.
